# An Assessment of Pulse Transit Time For Detecting Heavy Blood Loss During Surgical Operation

**DOI:** 10.2174/1874120701206010104

**Published:** 2012-12-28

**Authors:** Chien-Hao Wang, Cheng-Wei Lu, Tzu-Yu Lin, Maysam F Abbod, Jiann-Shing Shieh

**Affiliations:** 1 Department of Mechanical Engineering, Yuan Ze University, Taiwan, R.O.C; 2 Department of Anesthesiology, Far Eastern Memorial Hospital, Taiwan, R.O.C; 3 School of Engineering and Design, Brunel University, London, UK

**Keywords:** Pulse transit time (PTT), urologic surgery, cardiac surgery, blood loss, water supply.

## Abstract

The main contribution of this paper is the use of non-invasive measurements such as electrocardiogram (ECG) and photoplethysmographic (PPG) pulse oximetry waveforms to develop a new physiological signal analysis technique for detecting blood loss during surgical operation. Urological surgery cases were considered as the control group due to its generality, and cardiac surgery as experimental group since it involves blood loss and water supply. Results show that the control group has the tendency of a reduction of the pulse transient time (PTT), and this indicates an increment in the blood flow velocity changes from slow to fast. While for the experimental group, the PTT indicates high values during blood loss, and low values during water supply. Statistical analysis shows considerable differences (i.e., P <0.05) between both groups leading to the conclusion that PTT could be a good indicator for monitoring patients' blood loss during a surgical operation.

## 1 INTRODUCTION

The pulse transit time (PTT) is known as the time interval between the R-wave of the ECG and the start of the pulse wave in the periphery (for example at the fingertip) during a cardiac cycle. PTT was first introduced in the 1950s in psycho-physiological studies as an indication to anxiety and stress. Since the 1990s it has been used to measure sympathetic activation during upper airway obstruction during sleep. Recently, PTT has been used to monitor general or local anesthesia. Results of studies performed during general anesthesia indicate that PTT changes with anesthetic depth, while studies performed during general and spinal anesthesia suggest that PTT reflects autonomic tone and may function as a surrogate marker of arterial blood pressure [1, 2].

Furthermore, the ability of PTT to identity early stages of hypovolaemia has enormous benefits to clinical practice, in particular for cases associated with covert haemorrhage into body cavities that are not easily recognizable at early stages. Delayed control of abdominal, pelvic or intrathoracic haemorrhage has been recognized as a major contributor to preventable deaths trauma and is often caused by delays in the assessment or diagnosis of haemorrhage [3, 4]. Notably, it would be of great interest if such events could be detected as early as possible based on information that could be obtained from existing patient monitoring devices [5].

Maintaining hemodynamic stability is crucial to guarantee patient safety during an operation and can reduce preoperative morbidity and mortality. However, unpredictable complication such as bleeding may occur even under the surveillance of clinicians. The impact of blood loss on patients depends on the volume of blood loss and how early it is detected. Unrecognized severe hemorrhage may result in death. Traditional standard hemodynamic monitoring includes blood pressure measurement and electrocardiogram (ECG). Such monitoring methods are non-invasive but insensitive to blood loss. On the other hand, hemodynamic monitoring methods are sensitive to blood loss, however, methods such as real-time arterial catheterization and central venous catheterization are invasive and can cause damage potentially. Several recent studies have found non-invasive PTT method as a reliable technique that can be derived from photoplethysmographic (PPG) pulse oximetry waveform and ECG to indentify blood volume changes in awake healthy subjects [6]. The purpose of this paper is to utilize the PTT non-invasive measurement such as ECG and PPG pulse oximetry waveforms for creating reliable indicator to blood loss during surgical operation. 

## 2 METHOD

PTT, measured as the interval from the R wave of ECG signal to the pulse plethysmograph upstroke, was used recently to assess cardiovascular responses to anaesthesia and intubation. The R wave of ECG signal is often used as proximal timing point because it is simple to detect and tolerant to motion artifact. Both ECG and plethysmograph waves can be obtained noninvasively using standard monitoring equipment. The basic concept of PTT is to measure the time interval of the arterial pulse wave between the vascular path lengths at two selected sites. However, for ease of measurement, the R-wave of the ECG has been used as the starting point as it corresponds approximately to the opening of the aortic valve. Advances in technology have allowed accurate estimation of the arrival of the pulse wave at a peripheral site such as the fingertip or toe using PPG [7-9].

Using ECG signal and finger photoplethysmography to produce PTT measurements can be made very simple [10]. The derivation of oxygen in the blood is based on optical measurement of a peripheral volume pulse waveform, termed as the PPG pulse oximetry waveform, but its clinical significance has not been appreciated. Chan *et al.* [5] have demonstrated the possibility of monitoring variation in central blood volume using the finger photoplethysmogram and ECG. It is a difference of time. Indeed PTT is the time necessary for the blood to reach the PPG sensor from the heart (ECG), for one pulse as shown in Fig. (**1**). The unit to describe PTT is seconds [6, 7, 11, 12].

### Software Procedure to find PTT Step by Step:

To find PTT, first the consistency of the sampling time should be checked. That means the sampling time of ECG and PPG’s data should be the same. If the sampling instances are not the same, the program would stop right away. PTT is the time of arterial pulse wave transmitted between two arterial sites which offers beat-to-beat vascular information. Each arterial pulse wave begins with each contraction of the heart, and ends by the pulse wave travels to the terminal branches of arteries [13, 14]. The initial time can be obtained easily by the R wave of the ECG. The terminal time can be taken by the wave of PPG pulse on the fingertip. PTT should be always available since the use of ECG and PPG equipments are the mandatory during any kind of anesthesia [10, 15-16]. In order to calculate the PTT value, the maximum point on the ECG’s signal has to be identified first. Finite difference method can be used to identify all the maxima points on the ECG signal. During surgeries, the ECG signal can be contaminated by noise due to patient’s body movements, electric noise (i.e. diathermy effect) making the peaks detection difficult. In such cases, the use of filters such as period threshold or amplitude threshold can be effective. The period threshold filter checks if values from previous step are less than 300 ms or higher than 1500 ms which is unusual heart rate for people. While the amplitude threshold filter uses sliding window for deleting the RR interval. Though the sliding window’s step and length can be changed, an optimal parameter value of the sliding window should be found via trial-and-error according to surgery type. The sliding window size is required to be choosen not too wide or too narrow. If it is too wide, it will include two R peaks. However, if it is too narrow, it cannot find the R peak. The best situation is to find an R peak for every sliding window size. Since the sampling rate of ECG is 300 Hz, the sliding window size was set to 100 points which is about 333 ms in this study. Through numerous data analyses, 100 points for the sliding window is found to be the best setting in this study which is suitable for most patients. However, for some patients, the sliding window size had to be slightly adjusted to guarantee the detection of the R peak. An example for a clinical patient data of PTT measurement is shown in Fig. (**2**). Fig. (**3**) shows the results after using the filters. Finally, the calculation procedure flowchart is shown in Fig. (**4**).

## 3 EXPERIMENTS

### Equipment and Data Collecting Process

After obtaining the local hospital institutional ethics committee approval and written informed consent from the patients in the Far Eastern Memorial Hospital, data were collected from 25 urology surgery patients for control group and 5 cardiac surgery patients for experimental group. Patients were excluded if contraindications to ANH were found (hemoglobin <10 g/dL, age >70 yr, severe coronary artery disease, restrictive or obstructive lung disease, renal disease, or liver disease). AS/5 anesthesia machine and a notebook were used to collect the data. AS/5 is a multi-function physiological monitor that can measure the patient’s physiological signals such as BP, ECG, respiration and PPG, in real time. 

### Experiment for Control Group

For the control group, the patients’ data were collected from urological surgery. The patients who participated in this study are aged 15 to 65. All the patients had general anesthesia. As the control experiment, the patients’ data were logged before and during the surgical operation.

### Experiment for Experimental Group 

For the experimental group, it is intended to test the tendency of the PTT to vary when there is blood-loss or water supply. In this situation, cardiac surgery was selected for the experimental group because patients are required to have blood loss and water supply before the operation starts. In the operation, patients would have a step that can reduce blood loss and blood transfusion called acute hemodilution. When loosing blood, the hematocrit gets lower. Therefore, for the same amount of blood loss, the patients who have this step would lose less red blood cells. The other advantage is that the blood can transfer back to the patients themselves. It can reduce allergenic blood.

In order to detect the change of PTT, the patients have blood loss and water supply before the operation in this experiment. To determine the state of the patient during the experiment, the experiment process is divided into three stages as shown in the left part of Fig. (**5**). The three stages include the pre-operative preparation, experiment and operation start, and the transition process (arrow) between adjacent 1st and 2nd stage is induction. In the right part of Fig. (**5**), after induction, the experiment starts. The experiment is separated into two substages. During the first substage, the patient lost blood suddenly, and then was supplied with water in second substage.

### Statistical Analysis

The values are expressed as the mean±SD. The data obtained from two groups were compared using the paired Student’s *t*-test. A *P* value of less than 0.05 was considered significant [17].

## 4 RESULTS

### Control Group Results

**Fig. (6):**
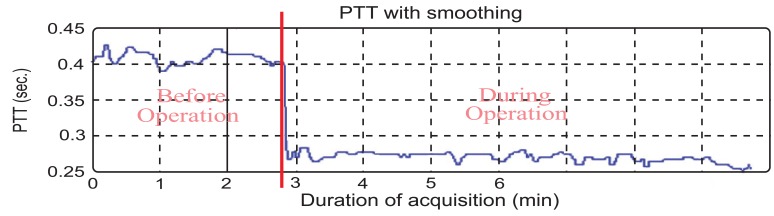
The PTT during urological surgical operation.

The results of the control group show the PTT during urological surgery. In this surgery, the patient did not have blood loss and water supply. The PTT average before the operation started is 0.3470±0.0412s and during the operation is about 0.2463±0.0271s as shown in Table **1**. It can be observed that the blood flow during the operation is faster than before the operation started (Fig. **6**). Statistical analyses were used to compare these two samples. It was found that *P*<0.0001, which means that the two samples have significant difference. According to anesthesiologists’ clinical experience, the patients are first injected with the quick anesthesia drug (propofol), however this narcotic drag is painful for the patient. For this reason, anesthesiologists always inject another narcotic drag (alfentanil). This drug could burst heart and cause the result.

### Experimental Group Results

The results of this experiment show the variation of PTT during three stages (i.e., pre-operative for stage 1, experiment for stage 2, and operation start for stage 3) and two substages (i.e., blood loss for substage 1 and water supply for substage 2) as shown in Fig. (**7**). The average of PTT during stage 1 is 0.2895±0.0261s, blood loss is about 0.3148±0.0212s and water supply is 0.2792±0.0126s as shown in Table **2**. Statistical analysis was used to compare these three stages. It can be observed that the first and second stages have significant difference (*P*<0.05). Furthermore, the second and third stages have significant difference (*P*<0.05). This result means that all the stages have significant difference. Analysis show that PTT in stage 2 is highest, which means the patient’s blood flow is the slowest during the blood loss process. During the process of blood loss, the patient will lose suddenly a great amount blood. In this situation, the heart cannot endure, so it might cause the blood flow to balance slowly. During stage 3, PTT is the lowest, which means that the patient’s blood flow is the fastest during the water supply process. According to anesthesiologists’ clinical experience, the concentration of the blood would be diluted during the process of water supply. That is the reason why PTT become lower during water supply.

The control group results show the PTT tendency to change from high to low. It meant that the velocity of blood flow was changed from slow to fast during an ordinary operation as shown in Fig. (**8**). While for the experimental group, it can be seen that during the three stages the PTT was first low, then became high during blood loss, and then low again during water supply as shown in Fig. (**9**).

## 5 DISCUSSIONS AND CONCLUSIONS

In this paper, it has been demonstrated that PTT is a good index for blood loss during surgical operation. However, the PTT calculation algorithm has some parameters setting which are still based on trial-and-error, in particular the sliding window size. Although most of patients cases were analysed using a single value, further investigations are still required so that an adaptive threshold can be achieved [18, 19] to meet higher detection accuracy for noisy ECG signals. Further investigations are required to test the methodology in detecting small blood loss. However, this can be seen from previous papers [5-6, 20] which have used blood donation for research. Although the amount of blood loss during blood donation is relatively small, it is sufficient to cause a reduction in circulatory blood volume and it has been shown that the changes of PTT over time may still be useful for detecting ongoing blood loss in the initial phase. Similarly, it can be seen that PTT might be a good index for detecting small blood loss using in surgical operation which so far having nothing reported in the field. Therefore, this finding can be applied in surgical operation to detect blood loss. Clinically, it is difficult for surgeons or anesthetists to estimate the amount of blood loss when monitoring the sucking of fluid from patients, which is a combination of blood and water. Furthermore, such event can become a vital problem if the blood loss happens inside body without being detected. This study demonstrates that PTT can be a good index using during surgery, which can help medical doctors to know better the physiological situations of their patients.

## Figures and Tables

**Fig. (1):**
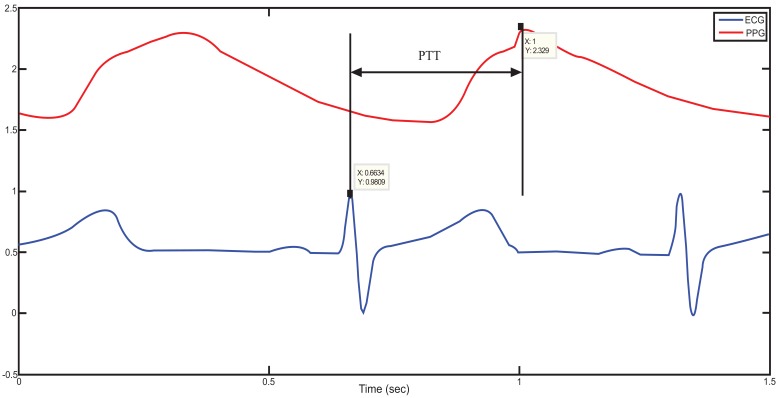
The definition of the PTT from a real patient data.

**Fig. (2):**
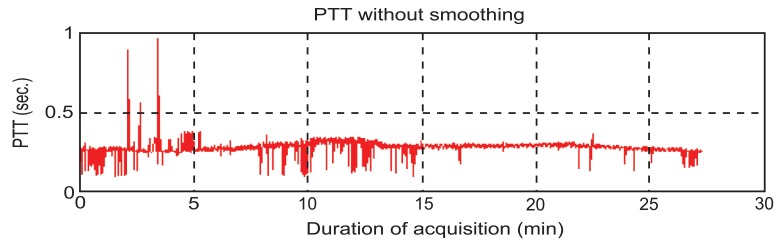
The original PTT’s curve.

**Fig. (3):**
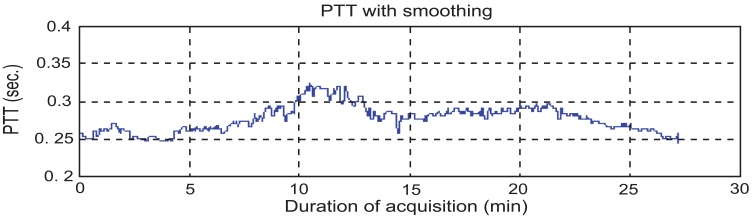
PTT’s curve after smoothing.

**Fig. (4):**
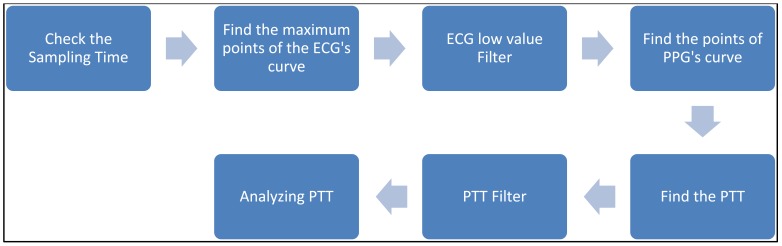
Finding PTT by software.

**Fig. (5):**
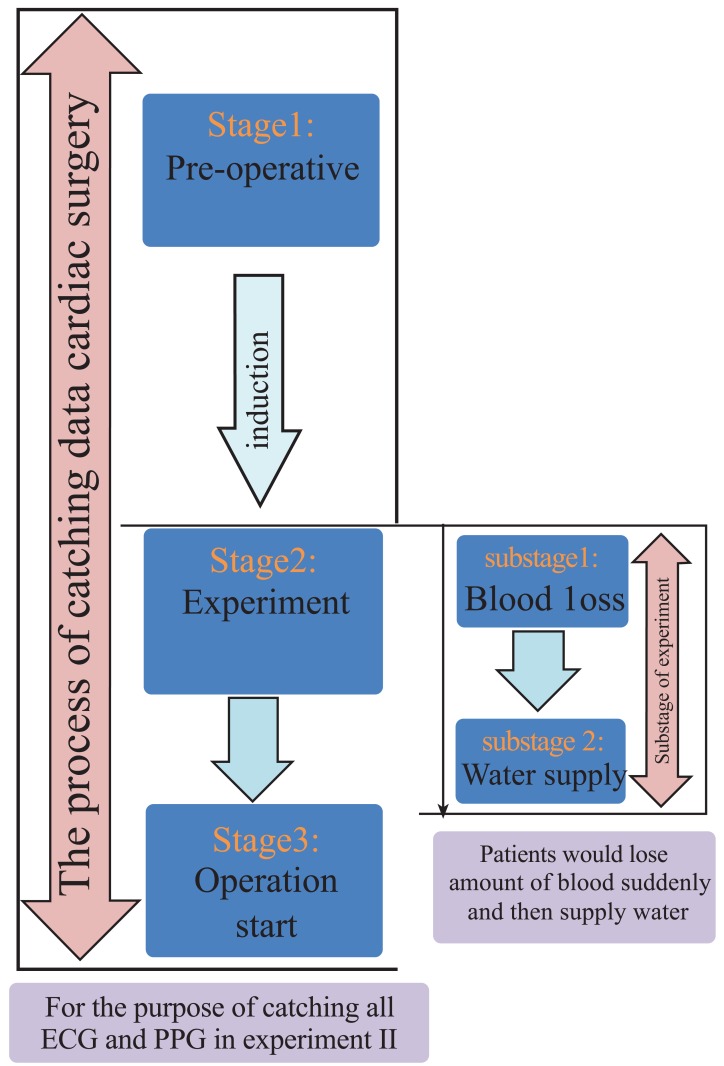
Flow chart of experimental method including the 3 stages of the process catching data in surgical operation and two substages in this experiment.

**Fig. (7):**
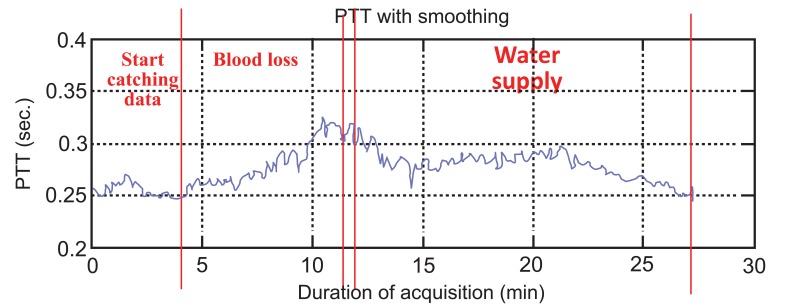
The PTT during cardiac surgical operation.

**Fig. (8):**
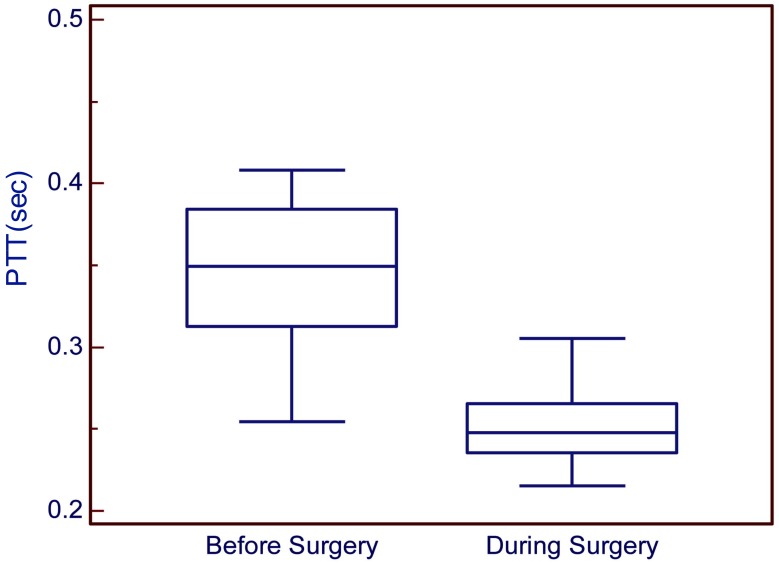
Comparing the two stages for the control group.

**Fig. (9):**
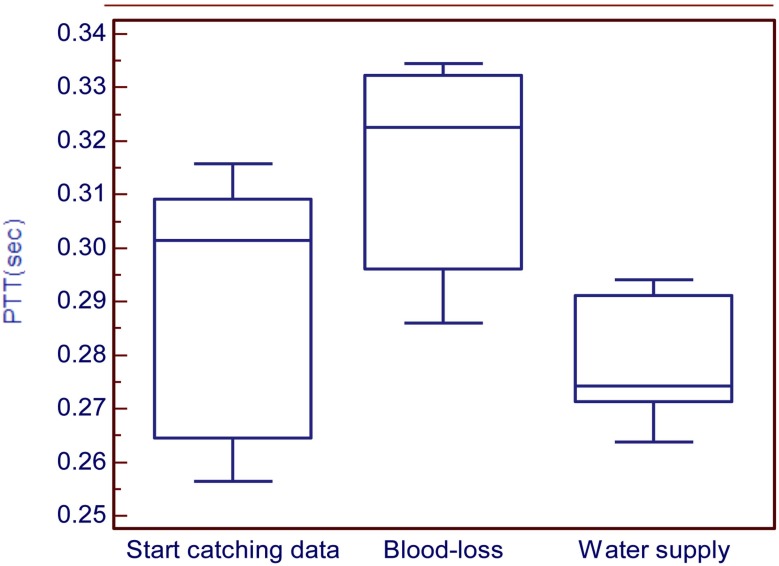
Comparing the three stages for the experimental group.

**Table 1:** The PTT in the Experiment I

No.	Gender/Age	Anesthesia	Before Surgery	During Surgery
1	F/58	General	0.3026±0.0320	0.2287±0.0327
2	F/43	General	0.3003±0.0026	0.2345±0.0250
3	M/40	General	0.3866±0.0315	0.2686±0.0084
4	F/58	General	0.3375±0.0380	0.2245±0.0088
5	F/54	General	0.3049±0.0161	0.2242±0.0067
6	M/57	General	0.3876±0.0091	0.3053±0.0169
7	M/44	General	0.3449±0.0241	0.2482±0.0100
8	F/50	General	0.3091±0.0258	0.2444±0.0050
9	F/48	General	0.4083±0.0087	0.2693±0.0129
10	F/57	General	0.3837±0.0092	0.2496±0.0050
11	F/53	General	0.4052±0.0229	0.2625±0.0261
12	F/61	General	0.3137±0.0139	0.2156±0.0099
13	F/51	General	0.3212±0.0192	0.2741±0.0113
14	F/33	General	0.3159±0.0157	0.2465±0.0147
15	F/60	General	0.4015±0.0216	0.2367±00235
16	F/51	General	0.3496±0.0148	0.2658±0.0163
17	F/62	General	0.3045±0.0368	0.2514±0.0270
18	F/55	General	0.2547±0.0374	0.1587±0.0220
19	F/41	General	0.3542±0.0653	0.2476±0.0173
20	F/53	General	0.4065±0.0145	0.2711±0.0891
21	M/42	General	0.3576±0.0560	0.2536±0.0132
22	M/51	General	0.3251±0.0148	0.2658±0.0175
23	F/57	General	0.3642±0.0162	0.2471±0.0034
24	F/34	General	0.3598±0.0263	0.2254±0.0074
25	F/42	General	0.3751±0.0082	0.2389±0.0358
Average ± SD			0.3470±0.0412	0.2463±0.0271

Note: Before operation vs. during operation, *P*<0.0001

**Table 2:** The PTT in the Experiment II

No.	Anesthesia	Start Catching Data	Blood Loss	Water Supply
1	General	0.3157	0.3344	0.2902
2	General	0.3015	0.3225	0.2638
3	General	0.2565	0.2860	0.2737
4	General	0.2671	0.2995	0.2742
5	General	0.3069	0.3316	0.2941
Average ± SD		0.2895±0.0261	0.3148±0.0212	0.2792±0.0126

Note: Start catching data vs. blood loss, *P*<0.05; Blood-loss vs. water supply, *P*<0.05.
